# The dual role of polyethylene glycol-simulated drought stress in *Stevia rebaudiana* (Bertoni): growth reduction and integrated physiological, biochemical, and steviol glycoside responses

**DOI:** 10.1007/s12298-026-01807-2

**Published:** 2026-07-31

**Authors:** Shafiq Hussain, Mehran Ali, Muhammad Zahid, Simone Ribeiro Lucho, Caroline Dambroz, Joyce Dória

**Affiliations:** 1 Department of Agriculture, Federal University of Lavras (UFLA), P.O. Box 3037, Lavras, 37200-900 Brazil; 2 Department of Agrochemistry, Federal University of Lavras (UFLA), P.O. Box 3037, Lavras, 37200-900 Brazil; 3 Department of Botany, Federal University of Pelotas, Capao Do Leao Campus, Capao Do Leao, 96160-000 Brazil

**Keywords:** Drought stress, Secondary metabolite, Antioxidant enzymes, Steviol glycosides (SGs), *Stevia rebaudiana*

## Abstract

*Stevia rebaudiana* Bertoni is a globally recognized natural sweetener owing to its zero-calorie steviol glycosides (SGs). However, drought stress, one of the leading abiotic constraints to crop productivity, adversely affects stevia growth and development, while simultaneously altering its biochemical composition. This review highlights the impact of polyethylene glycol (PEG)-induced drought stress on stevia, with particular emphasis on agronomic traits, metabolites, antioxidant activities, and SGs biosynthesis. PEG-mediated stress reduces key growth parameters, such as plant height, biomass, and leaf area, but in some cases, it promotes root elongation as an adaptive strategy to optimize water uptake. Such alterations are accompanied by biochemical adjustments, notably the up-regulation of antioxidant enzymes, such as superoxide dismutase (SOD), peroxidase (POD), and catalase (CAT), which neutralize reactive oxygen species (ROS)-induced oxidative stress. Moreover, PEG stress enhances the accumulation of secondary metabolites, such as phenolic, flavonoids and terpenoids, thereby improving antioxidant potential and stress tolerance. Notably, PEG-induced drought stress shapes SG biosynthesis through the up-regulation of the methylerythritol phosphate (MEP) pathway and the differential expression of key SG-related genes. Such modulation frequently increases SG accumulation, thereby strengthening their medicinal and economic value. Overall, this review covers the dual nature of PEG-induced drought stress: while limiting agronomic performance, enhances SGs biosynthesis and antioxidant capacity. Gaining insights into these mechanisms will facilitate the development of optimized cultivation strategies under water deficit conditions. Future research should prioritize molecular breeding and advanced biotechnological approaches to enhance drought tolerance and maximize the production of bioactive metabolites in stevia.

## Introduction

*Stevia rebaudiana* Bertoni, commonly known as ‘stevia’, is a perennial shrub native to the Amambay area of Paraguay, South America, and belongs to the family Asteraceae (Verma 2024). It is particularly valued for its high content of steviol glycosides (SGs), natural zero-calorie sweeteners that have attracted global attention as an alternative to artificial sweeteners, especially in health-conscious markets (Muñoz-Labrador et al. 2024). Traditionally, the Guarani tribe of Paraguay was among the first to use stevia, referring to it as "Ka’a he’ê" means sweet herb. Earlier, stevia was used to add taste to beverages, such as mate, or for medicinal purposes, such as heartburn and diabetes (Wazir et al. 2025). Stevia research traces back to the last decades of nineteenth century when chemists first determined its sweetening properties. Today, stevia is grown globally, with the primary producers being China, India, and some parts of South America (Formigoni et al. 2021).

The sweetening compounds in stevia are SGs, primarily stevioside and rebaudioside A, which are estimated to be 200–300 times sweeter than table sugar (Okonkwo et al. 2024). Unlike many artificial sweeteners, SGs are heat-stable and have the potential to retain their functionality across a wide pH range, making them ideal for various food and beverage products (Benucci et al. 2025). These unique characteristics have accelerated the global adoption of stevia as a natural and renewable alternative to synthetic sugars (Peteliuk et al. 2021). From an economic perspective, stevia cultivation requires low inputs, while consumer demand continues to rise, driven by increasing awareness of the health risks associated with high sugar intake and the preference for natural flavoring agents. Thus, the global stevia market has developed significantly, with a reported compound annual growth rate (CAGR) exceeding 8% in the last few years, mainly attributed to consumer knowledge of the effects of high sugar content and the quest for natural flavors in foods (Perkasa and Mulyana 2024).

**Fig. 1:**
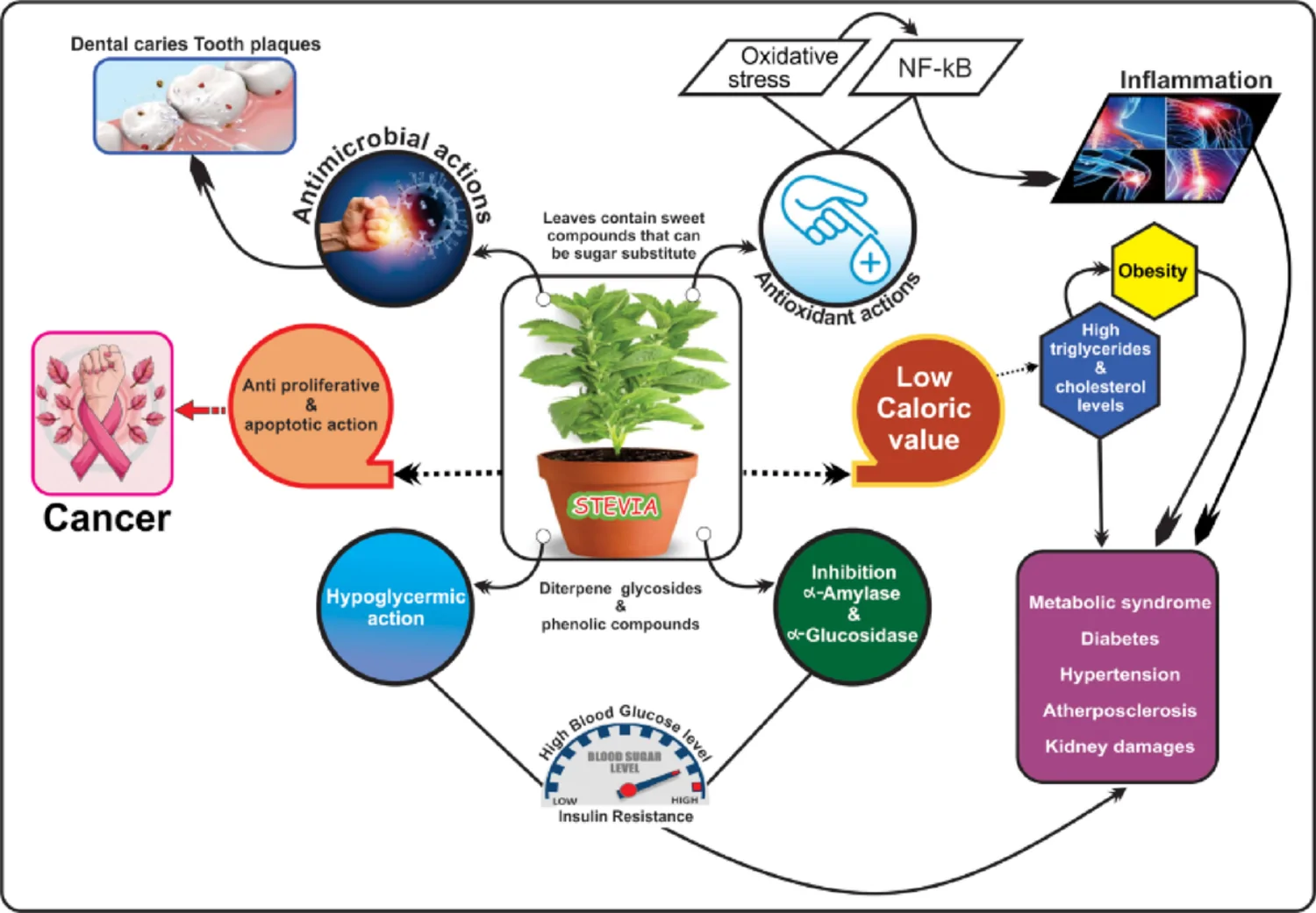
Schematic overview of the major health-promoting activities of *Stevia rebaudiana* leaf extracts and their bioactive constituents

Beyond that, stevia exhibits a wide range of medicinal properties, as shown in Fig. 1. It has been reported by clinical research that stevioside can lower hypertensive patients blood pressure since it helps widen blood vessels (Villegas Vílchez et al. 2022). It also increases insulin levels and promotes the utilization of glucose, thereby making stevia a promising therapeutic candidate for the management of type 2 diabetes mellitus (Jan et al. 2021). Additionally, stevia leaf extracts have antimicrobial activity and hinder the growth of bacteria, such as *Staphylococcus aureus,* and fungi, such as *Candida albicans* (Ibrahem et al. 2020). Moreover, high levels of phenolics (Eliwa and Ahmed 2025) in stevia contribute to the reduction of oxidative stress by scavenging free radicals and protecting cells from damage (Ameer et al. 2020). Collectively, these medicinal properties, together with its non-caloric nature, underscore the potential of stevia as both a functional food and a medicinal ingredient.

Besides that, stevia offers prominent sustainability advantages (Sanabria-Velazquez et al. 2025). It requires relatively less water and space than conventional sugar crops such as sugarcane and sugar beet (Abouelela et al. 2025), its adaptability to shallow and low-fertility soils and compatibility with organic cultivation further support its role in sustainable farming systems (Clemente et al. 2021). In contrast, stevia cultivation has some constraints, including its susceptibility to abiotic stresses, such as drought, high salinity, wind, fertilizers, and temperature fluctuations (Modi 2025; Chou et al. 2025). Notably, light is considered to be one of the important factors affecting growth and metabolism of a plant (Wu et al. 2025). Although stevia is a short day plant, it prefers sunny conditions for cultivation (Jarma-Orozco et al. 2020); however, the optimum light intensity which induces SG production is still controversial (Yoneda et al. 2017). Furthermore, it has been documented that light quantity and quality notably impact the regulation of both primary and secondary metabolism of the plant, and studies have previously evidenced that light conditions modulate the biomass production of stevia, secondary metabolites production, SGs accumulation, and gene expression involved in GA biosynthesis (Yang et al. 2018; Semenova et al. 2024). Beyond these effects, a study has reported that exposure to various UV ranges altered chlorophyll fluorescence indices, whereas UV-C irradiation promoted the light stress and exhibited reduced photosynthetic efficiency (Fv/Fm), indicating photoinhibition and growth inhibition (Semenova et al. 2024). Moreover, temperature also influences SG biosynthesis in stevia, as several genes involved in SGs production, exhibited maximal transcriptional activity at 25 °C, however their expression was reported to be inhibited at both low (15 °C) and high (35 °C) temperatures (Yang et al. 2015), thereby suggesting that high temperatures may adversely affect the growth and germination as well as SG accumulation (Hossain et al. 2017). Understanding the physiological and biochemical responses of stevia to extreme conditions is critical for developing stress-tolerant cultivars. Polyethylene glycol (PEG) is commonly used to stimulate drought stress and provide valuable insights into the mechanisms of stress tolerance (Sajid et al. 2025; Bao et al. 2025). Therefore, considering the sweetening, economic, medicinal, and environmental benefits of stevia, it is increasingly recognized as a promising crop for future agriculture. Moreover, the increased consumer preference for natural sweeteners makes the comprehensive evaluation of the performance of stevia under abiotic stresses, especially drought, a vital necessity for the advancement of its production efficiencies and metabolite production. Notably, studies employing PEG-simulated drought stress in *Stevia rebaudiana* remain limited, highlighting a significant gap in controlling investigations of drought stress effects on steviol glycoside biosynthesis. Therefore, this review provides current insights into the physiological and molecular responses of stevia to PEG-induced drought stress. This study further elucidates how structural traits (morpho-physiological and biochemical) affect the response of stevia to PEG-simulated drought and outlines potential strategies to enhance drought tolerance. In addition, it describes the signaling pathways, role of hormones, and the connection between metabolic changes caused by stress exposure and plant survival mechanisms.

## Stevia response to PEG-simulated drought stress

PEG is widely regarded as a true osmotic agent because of its non-ionic inert nature (Parray et al. 2020), which effectively reduces water potential while avoiding phytotoxic effects on plants (Saepudin et al. 2017). It is a synthetic polymer that serves many applications in pharmaceuticals and biotechnology owing to its high water solubility and excellent biocompatibility (D’souza and Shegokar 2016). Furthermore, it is a widely used method for enzyme stabilization because it stops protein aggregation and enhances the functionality of biochemical assays (Zuma et al. 2022).

**Fig. 2:**
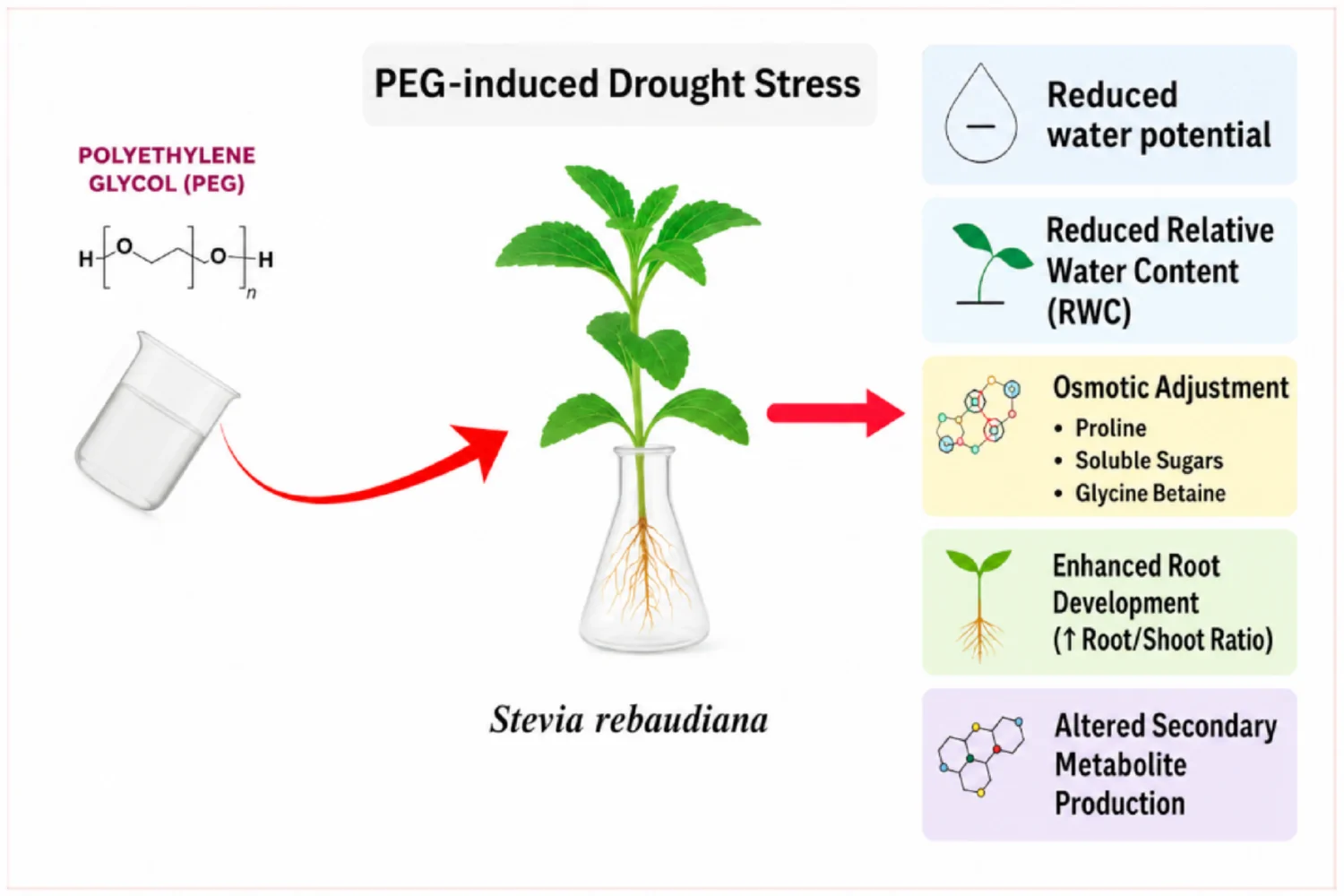
Stevia response to Polyethylene Glycol (PEG)-induced drought stress highlighting the reduction in water potential and relative water content (RWC), enhancement of root/shoot growth, and modulation of secondary metabolite profiles

In addition, PEG is widely used in plant physiology studies to stimulate drought stress through osmotic pressure without penetrating the cell wall of a plant, facilitating the evaluation of its effects on plant growth and metabolism, thereby providing insights into drought tolerance mechanisms (Bashir et al. 2021). Several studies have documented their roles in stevia plants. For example, Magangana et al. (2021); Hossain et al. (2024); Manokari et al. (2025) demonstrated that PEG-mediated drought stress lowers the water potential, simulating drought stress and allowing researchers to evaluate the impact of stevia under drought conditions. Likewise, it triggers various physiological alterations in stevia, including drought stress, minimizing the potential of cells to retain water, thereby reducing the relative water content (RWC) (Ghaderi et al. 2025), which consequently influences turgidity pressure and influences growth and cell expansion (Sheikhalipour et al. 2023). Upon exposure to drought stress, plants, including stevia, adopt adaptive strategies through osmotic adjustment, associated with the accumulation of compatible solutes including, total soluble sugars, glycine betaine, and amino acids i.e. proline, thereby helping to maintain cellular homeostasis (Ghorbani et al. 2025; Batista‐Silva et al. 2019; Timofeeva et al. 2024). It has been reported that proline plays diverse roles beyond its function as an osmolyte in osmotic balance. It also contributes to stabilizing cellular structures, maintaining cellular redox potential, and protecting the photosynthetic pigments (Afshari et al. 2022; Chen et al. 2022). To understand the mechanism, under drought stress, stevia closes its stomata to reduce water loss through transpiration (Guo et al. 2019; Dubey et al. 2023), which tends to lower the carbon dioxide (CO_2_) intake, adversely affecting plant photosynthetic functions and productivity (Agehara and Leskovar 2012). Furthermore, PEG stress may stimulate deeper root growth and increase the root/shoot ratio as adaptive responses, thereby enhancing the plant's ability to use water efficiently (Robin et al. 2021; Licaj et al. 2023). A range of studies have shown that stevia can elongate its roots under PEG-induced stress, indicating its potential to withstand drought conditions (Spinoso-Castillo et al. 2023; Khan et al. 2024; Ahmad et al. 2020; Hajihashemi and Sofo 2018). PEG-induced drought stress has also been shown to promote the secondary metabolite production in stevia plants (Ghazal et al. 2024). As an alternative to PEG, salinity stress can also be used to stimulate stress conditions in stevia. Unlike PEG stress, salt stress imposes both osmotic and ionic stresses, leading towards different physiological and metabolic responses. Stevia is considered as a moderately salt tolerant species (Zeng et al. 2013). Studies have reported that stevia under excessive NaCl-mediated salt stress reduces the chlorophyll pigments and plant growth (Sharma et al. 2020), whereas, interestingly, mild stress (30 mM) exhibited an increase in SG accumulation (Cantabella et al. 2017; Ceunen and Geuns 2013a), possibly due to the plants osmotic adjustments under salinity stress. Thus, it may concluded that mild salt stress could serve as an alternative strategy to enhance the stress-responsive osmolytes. Taken together, PEG-induced drought stress generally inhibits the overall plant growth by limiting water availability. However, under moderate stress conditions, it triggers a range of adaptive responses that enhance stress tolerance (Rajeswar and Narasimhan 2021). Therefore, PEG serves as a reliable tool to understand how stevia copes with drought conditions, from root development to metabolic alterations, providing valuable insights into its drought tolerance mechanisms. Key responses of stevia to PEG stress are shown in Fig. 2.

## Influence of PEG on plant growth and development

**Fig. 3:**
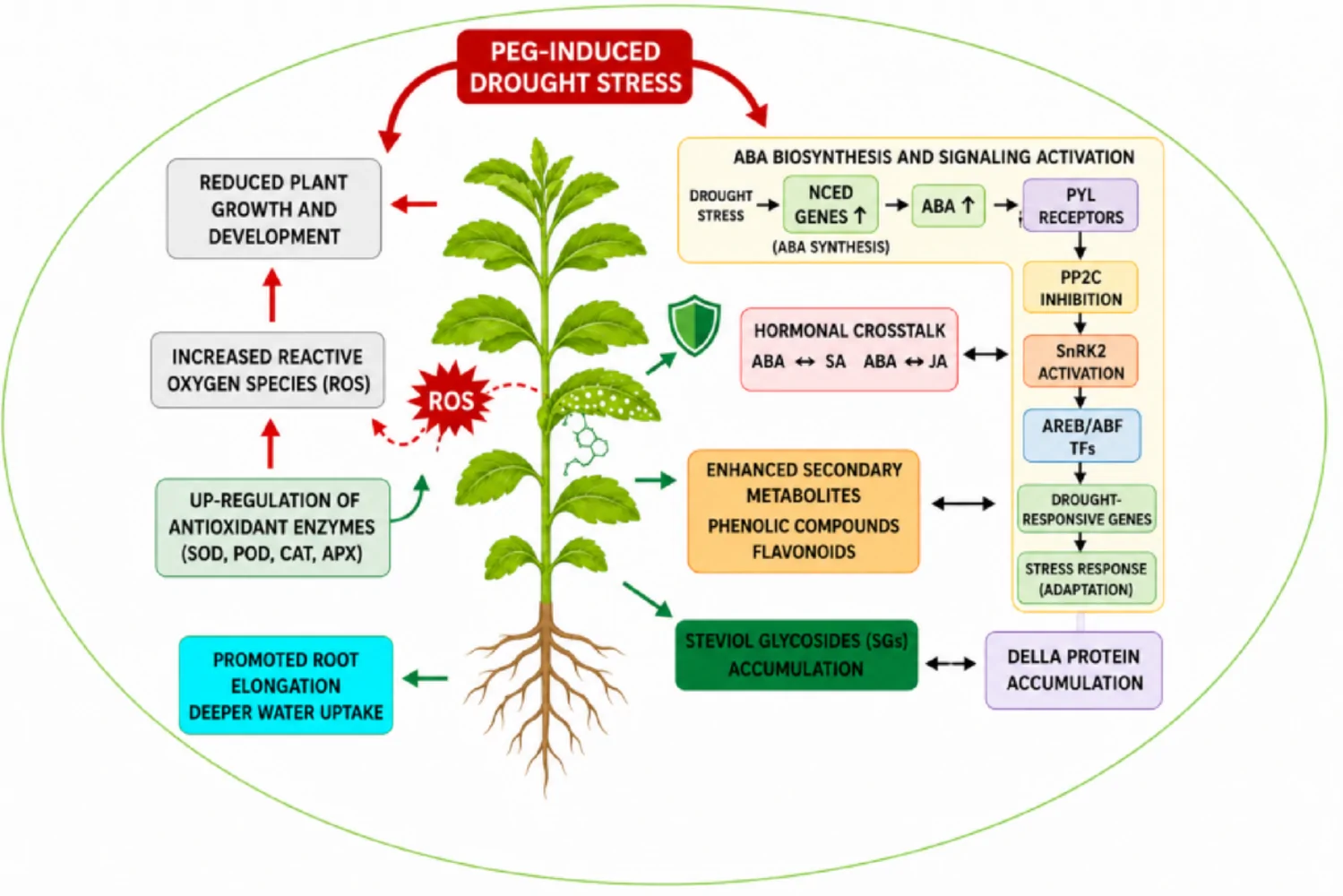
This figure represents the overall physiological and biochemical responses of *Stevia rebaudiana* to Polyethylene Glycol (PEG)-mediated osmotic stress

PEG-induced drought largely affects early stages of growth and development of stevia (Afshari et al. 2020) by reducing water availability and lowering seed water potential and uptake, thereby delaying germination (Hajihashemi and Sofo 2018). This limits nutrient availability, leading to reduced biomass accumulation in seedlings (García-Cánovas et al. 2024). It further reduces shoot and root biomass (fresh and dry), leaf area, and plant height (Megasari et al. 2025). Such decline in shoot biomass is often observed as the metabolic energy and resources are diverted towards root development and stress tolerance mechanisms (Afshari et al. 2022; Ahmad et al. 2020). Upon exposure to drought conditions, plant cellular activities become limited, leading to slower growth and development of smaller leaves with lower photosynthetic capacity (Qiao et al. 2024; Hemati et al. 2022). In addition, it alters root morphology, enabling deeper water penetration as an adaptation (Ghaderi et al. 2024). However, PEG adversely affects the reproductive phase of stevia, persisting vegetative growth, and delaying flowering (Pradhan et al. 2020). Moreover, PEG-modulated drought stress accelerates senescence, with leaves exhibiting chlorosis and necrosis, ultimately contributing to a reduced overall growth (Afshari et al. 2022). The influence of PEG-induced drought stress on stevia growth and development is illustrated in Fig. 3.

At the cellular level, PEG-induced drought stress disrupts key growth processes by increasing cell wall rigidity, thus restricting cell suspension and tissue development (Ashrita et al. 2024). It has been reported that, drought stress conditions elevate abscisic acid (ABA) levels while reducing gibberellic acid (GA), cytokinin (CK), and auxin levels, resulting in suppressed growth (Xu et al. 2024; Muhammad Aslam et al. 2022). ABA-related biosynthesis and signaling genes are overexpressed during drought stress for osmotic adjustment and stress adaptation. Drought stress, in particular, upregulates the ABA biosynthesis via 9-cis-epoxycarotenoid dioxygenase (*NCED*) genes encoding key enzymes responsible for ABA biosynthesis, essential for mediating the abiotic stresses like drought. ABA signaling is detected by PYL receptors, which inhibit PP2C phosphatases and activate sucrose non-fermenting-related protein kinase 2 (SnRK2) kinases. The activated SnRK2s subsequently phosphorylate downstream key transcription factors, including AREB/ABFs, thereby modulating the expression of various drought-responsive genes involved in osmotic balance, stomatal regulation, antioxidant defense, and stress tolerance (Zhang et al. 2025). In addition, ABA signaling exhibits extensive crosstalk with other phytohormonal pathways including salicylic acid (SA) and jasmonic acid (JA), which coordinates plant responses to abiotic and biotic stresses (Zahid et al. 2026). ABA implies the accumulation of DELLA proteins, which further inhibits growth-related processes and redirects resources toward stress adaptation, improving survival under stressful conditions (Das et al. 2025). Furthermore, it has also been documented that changes in the expression of stress-responsive genes affect cell division, elongation, and differentiations as well as hinder the overall development (Biswas et al. 2024). In stevia, PEG stress drastically reduces photosynthetic efficiency, leading to growth inhibition, mainly due to stomatal closure most of the time to avoid excess water loss, which hampers CO_2_ uptake and improper light absorption, promoting chlorophyll degradation, thereby affecting the photosynthetic rate (Pompelli et al. 2022; Rodriguez-Paez et al. 2023) (Table 1).

**Table 1:** Response of PEG-modulated drought stress on *Stevia rebaudiana.* Bertoni, upon exposure to different growth conditions

Plant growing conditions	Treatments	Influence of PEG stress	References
Grown in-vitro	Five treatments including; Control, 1%, 2%, 3%, and 4% (w/v)	Improved SOD, POD, CAT, and APX activities. Promoted secondary metabolites and increased SGs content	Khan et al. (2024)
Green-house study	6-month-old plants irrigated with 0% (distilled water), 5, 10, and 15% (w/v) of PEG	Reduces plant mass, photosynthesis, chlorophylls, and carotenoids. ROS accumulation and activity of some enzymatic and non-enzymatic antioxidants	Hajihashemi and Sofo (2018)
In-vitro condition	PEG applied for 4 weeks, i.e., 0, 0.5, 1, 2, and 4%	Production of secondary metabolites	Ahmad et al. (2020)
In-vitro condition	25 to 100 mM NaCl and PEG at 2.5% to 10.0% (w/v) to MS media	Decreased plant growth and development, as well as rebaudioside A and stevioside	Magangana et al. (2021)
Hydroponic condition	Used 2 genotypes, seven treatments of PEG (Control, -2, -4, -6, -8, -10, and -12 bars)	Both genotypes reduce yield and growth indices, however, drought stress (-2 bars) increased SGs contents compared to control	Ghaderi et al. (2024)
In-vitro condition	PEG and proline treatment in callus and suspension culture	PEG enhances SGs contents in both cultures	Gupta et al. (2015)
Greenhouse condition	Salicylic acid, sodium alginate, PEG, chitosan and yeast extract	Increases total SGs, positive correlation of SGs-related genes with the metabolite content	Ashrita et al. (2024)
In-vivo condition	Integrated PEG with salinity stress and waterlogging stress conditions	Gene expression analysis revealed marked up-regulation of ROS-detoxifying, osmotic-adjustment, ion-transport, and stress-responsive genes	Pal et al. (2023)
In-vitro condition	5% PEG, NaCl; (50 and 100 mM) and gibberellic acid (2.0 and 4.0 mg/L GA_3_)	HPLC analysis revealed significant biosynthesis of important steviosides, also found positive correlation between the elicitors	Ghazal et al. (2024)
In-vitro condition	PEG along with various elicitors (biotic and abiotic)	Enhanced rebaudiosides, particularly RA and RC content along with stevioside and its related genes	Thakur et al. (2021)

## Role of PEG in modulating antioxidant enzyme activity

To understand how PEG-mediated drought stress influences overall plant growth and productivity, it is essential to investigate its role in modulating stress-related responses in plants, including stevia, at the cellular level. One key response to such stress involves the activation of antioxidant enzymes, which play a critical role in protecting cells from oxidative damage caused by ROS generated during osmotic stress (Ashraf and Harris 2013). ROS interact with cellular macromolecules, including proteins, lipids, and nucleic acids, leading to membrane lipid peroxidation, protein disruption, and DNA damage (Torres-Franklin et al. 2008). Furthermore, ROS induces oxidative damage within chloroplasts, reducing carboxylation efficiency (Potters et al. 2010). In addition, other metabolic organelles, such as mitochondria and peroxisomes, contribute to ROS accumulation through electron transfer and photorespiration, respectively. This oxidative damage is commonly triggered by environmental stressors, such as drought, making the regulation of these enzymes vital for plant survival and resilience (Noctor and Foyer 1998).

### Enzymatic and non-enzymatic antioxidants

Upon exposure to drought stress, plants activate both enzymatic and non-enzymatic antioxidants, as well as osmolytes, including proline and glycine betaine, to cope with ROS-induced oxidative damage. They play a crucial role in ROS detoxification, mitigating cellular damage, and maintaining physiological homeostasis (Qi et al. 2023). Non-enzymatic antioxidants (metabolites), for example, ascorbate, glutathione, carotenoids, and phenolic compounds, also contribute to reducing the ROS levels in plant tissues (Torres-Franklin et al. 2008). Among the antioxidant enzymes, superoxide dismutase (SOD) is the first and primary response mechanism to combat oxidative stress in plants (Ighodaro and Akinloye 2018; Gill et al. 2015). SOD catalyzes the dismutation of superoxide radicals (O₂⁻) into hydrogen peroxide (H_2_O_2_) and molecular oxygen (O₂) (Alfei et al. 2024). Studies have revealed that exposure of stevia to PEG-induced drought stress leads to a significant increase in the production of ROS, such as superoxide radicals, which in turn stimulates the activation of SOD (Zheng et al. 2023; Melo-Sabogal and Contreras-Medina 2024; Hajihashemi and Ehsanpour 2014).

Furthermore, the H_2_O_2_ generated during SOD-catalyzed dismutation is subsequently detoxified by other antioxidant enzymes such as peroxidase (POD), catalase (CAT), and ascorbate peroxidase (APX), which convert H_2_O_2_ into H_2_O in different cellular compartments (Anjum et al. 2012). During drought stress, PEG enhances the generation of H₂O₂, thus requiring the activation of CAT to avoid oxidative damage (Kralova and Jampilek 2021). Moreover, APX plays a crucial role in maintaining cellular redox balance by reducing hydrogen peroxide (H₂O₂) to water through the oxidation of ascorbate (vitamin C) to dehydroascorbate. This reaction is essential for preventing the excessive accumulation of H₂O₂, a major contributor to oxidative damage under stress conditions (Corpas et al. 2024). In *S. rebaudiana*, PEG-induced drought consistently increased APX activity, reflecting the plant’s response to increased ROS formation. The enhanced activity of APX supports more efficient H₂O₂ detoxification and contributes to improved oxidative stress tolerance, thereby reinforcing the plant capacity to withstand drought-related metabolic disruptions (Petrova et al. 2024).

**Fig. 4:**
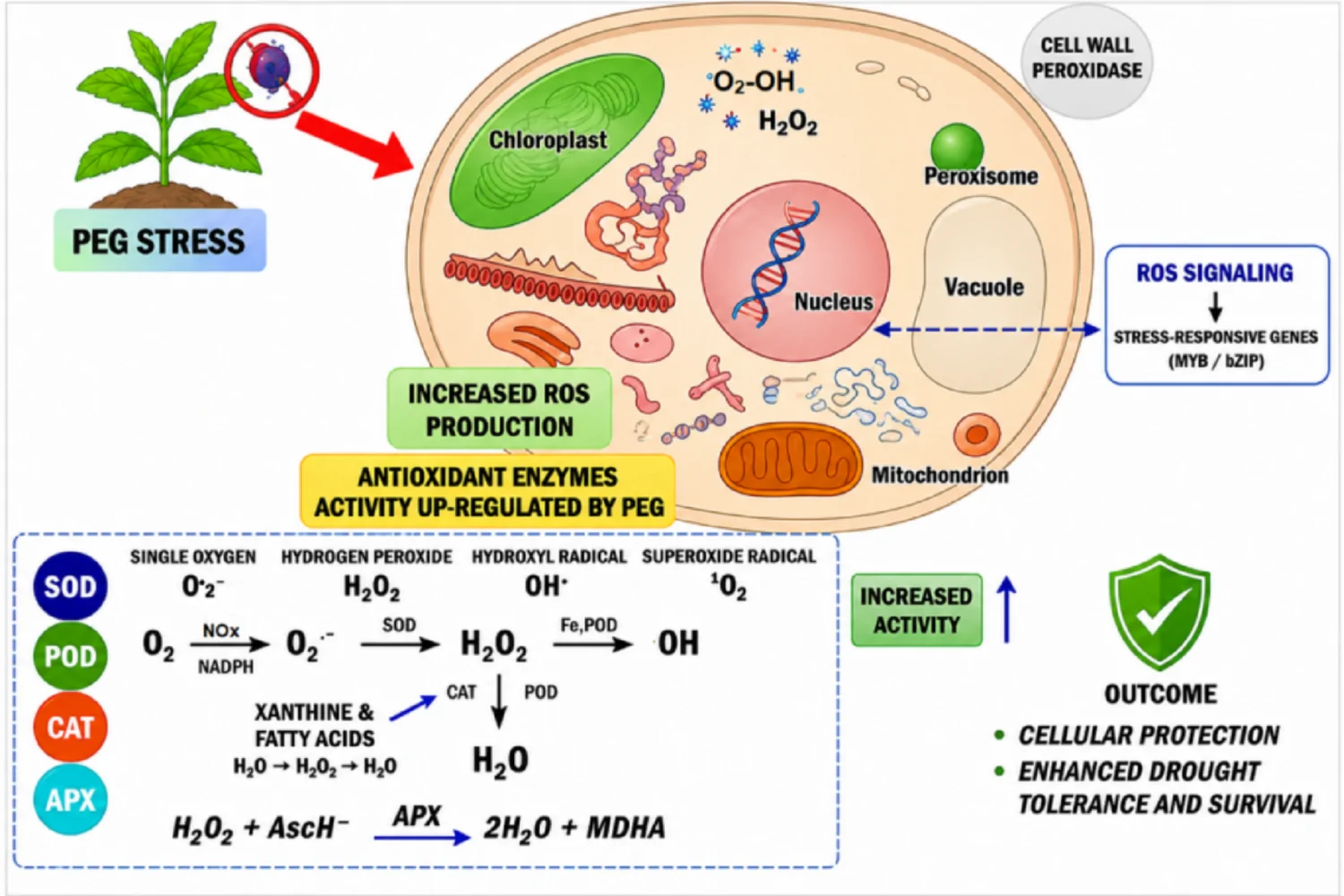
This schematic representation illustrates Polyethylene Glycol (PEG)-induced drought stress-mediated reactive oxygen species (ROS) production and the activation of antioxidant enzymes in *Stevia rebaudiana* through specific pathways

In addition, polyphenol oxidase (PPO) contributes to oxidative defense by catalyzing the oxidation of phenolic compounds, thereby regulating the cellular phenolic concentration in plants under stress conditions (Hajihashemi and Ehsanpour 2014). A number of studies have highlighted the importance of phenol-oxidizing enzymes in plant responses to drought stress (Veljovic-Jovanovic et al. 2006; Xu et al. 2011; Živković et al. 2010). Furthermore, prior studies conducted on a range of plant species, including stevia, have revealed noteworthy patterns in antioxidant responses under PEG-induced drought stress. Most studies have highlighted enhanced antioxidant responses and adaptive stress defense mechanisms during PEG stress in stevia, whereas growth parameters are generally reduced under stress conditions (Hajihashemi and Geuns 2016; Hajihashemi and Sofo 2018; Hajihashemi et al. 2018; Gorzi et al. 2018; Villalba and Nakashima 2016). For instance, Hajihashemi and Ehsanpour (2014) observed a progressive increase in the antioxidant activities of stevia with increasing PEG concentrations (0, 2, 4, and 6%). Similarly, Hajihashemi and Sofo (2018) noted a reduction in growth indices while documenting an increase in antioxidant enzymes in stevia having (5, 10, and 15%) of PEG concentrations. Conversely, Pradhan et al. (2020) documented a decline in antioxidant activity under PEG-induced drought stress both in vitro and in vivo, indicating that stevia responses may vary depending on the developmental stage, stress severity, and culture conditions. In summary, the evidence underscores the crucial role of antioxidant activity up-regulation in enhancing drought tolerance in stevia and protecting it against over-production of ROS-induced oxidative stress (Fig. 4).

## PEG effects on secondary metabolite profiles and their pathways in *Stevia rebaudiana*

Plants rely largely on a diverse range of secondary metabolites, mainly phenolics, flavonoids, and terpenoids, which act as key defensive compounds to help plants cope with various environmental stresses (Kumar et al. 2023). They play an important role in buffering oxidative damage, especially under drought stress, where severe ROS are generated. PEG is commonly used to simulate drought stress in experiments, modulates the biosynthesis of these compounds in stevia. Studies reported that PEG treatment enhances the production of phenolic and flavonoid contents, thereby reinforcing the plant’s protective capacity and contributing to improved drought stress tolerance (Magangana et al. 2021; Abbas et al. 2023). Phenolic compounds and flavonoids are among the most effective non-enzymatic antioxidants, capable of scavenging the overproduction of ROS under stress conditions. It is indeed a fact that, excess amount of ROS can damage proteins, lipids, and DNA, ultimately disrupting cellular function (Krasensky and Jonak 2012). Similar to other plant species, *Stevia rebaudiana* responds to drought stress with increased synthesis of secondary metabolites as part of its protective strategy (Ghazal et al. 2024). Notably, PEG-induced drought stress promoted total phenolic content by approximately 30–34% and flavonoid levels by 24–29%, demonstrating a substantial metabolic adjustment that enhances oxidative stress tolerance in stevia (Ahmad et al. 2020).

Furthermore, higher accumulation of phenolics and flavonoids under PEG stress helps in reducing oxidative damage and contributes to the enhanced medicinal efficacy of stevia. These compounds have the potential to stimulate the therapeutic effects of plant in terms of anti-inflammatory, antioxidant, and antimicrobial uses (Ghazal et al. 2024). Terpenoids are another group of secondary metabolites that act as signaling molecules and serve in stress-related pathways. Importantly, they modulate the expression of genes involved in plant defense mechanisms (Alam 2021). The increase in terpenoid levels under PEG stress reflects the activation of stress-responsive metabolic pathways in *Stevia rebaudiana*. This metabolic adjustment is often accompanied by the up-regulation of antioxidant defense genes, including SOD and CAT, which together limit the over-expression of ROS and ultimately mitigate oxidative damage to stevia. Thus, it is considered an integral component of the plant’s defense strategy, contributing to both ROS scavenging and the stabilization of cellular functions under stress scenarios. Previous studies by Pal et al. (2023) and Shahnawaz et al. (2021) demonstrated that water-deficit stress significantly stimulates terpenoid accumulation, emphasizing their critical involvement in plant stress tolerance and their potential contribution to the enhanced pharmacological and therapeutic properties of stevia.

PEG-induced drought stress enhances the antimicrobial potential of *S. rebaudiana*. The secondary metabolites accumulated during PEG stress, particularly terpenoids and flavonoids, exhibit notable antibacterial activities, further suggesting that drought-triggered metabolic modulation may strengthen the defensive and bioactive properties of stevia (Abdelsattar et al. 2023). Stevia contains a smaller number of alkaloids compared to other plant species, such as stevianine, which tends to be produced during drought stress and exhibits antimicrobial activity against diseases caused by various pathogens (Gupta et al. 2015). Lignans, phenolic metabolites derived from the phenylpropanoid pathway, are stress-responsive compounds in many plant species. In *S. rebaudiana*, these are presumed to contribute to antioxidant defense and limit oxidative damage. Although direct evidence for PEG-induced lignan accumulation in stevia is limited, findings from related species suggest that these compounds may play an essential role in enhancing drought tolerance (Samal et al. 2023).

In addition to the isolated action of PEG, a promising area of research explores the co-application of PEG with other elicitors to further modulate the secondary metabolite profile. Studies such as Ashrita et al. (2024) have investigated integrating PEG with substances like SA, sodium alginate, and chitosan. These multi-elicitor approaches can lead to synergistic effects, activating broader or distinct signaling pathways that, together, upregulate metabolite biosynthesis more effectively than individual treatments. Conversely, antagonistic interactions are also possible, highlighting the need for careful optimization. Understanding this interaction is crucial for the development of sophisticated strategies that optimize the yield and profile of the desired secondary metabolites in stevia, opening new perspectives for enhancing its medicinal and economic value.

**Fig. 5:**
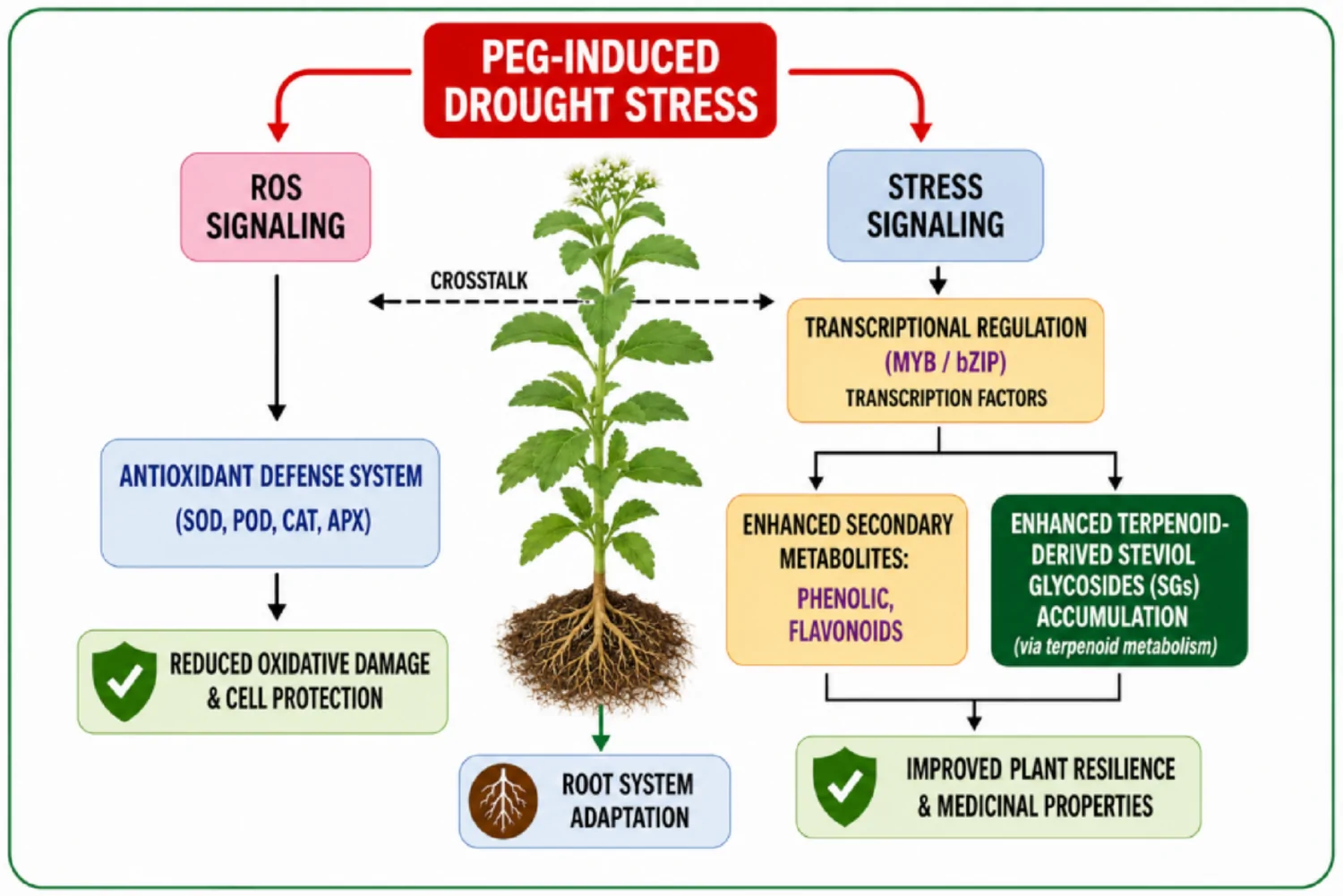
Integrated view of Polyethylene glycol (PEG)-mediated reactive oxygen species (ROS) generation and stress signaling in *S. rebaudiana*

A relatively new area of investigation includes epigenetic mechanisms, such as DNA methylation, histone modification, and miRNA-mediated regulation, which play an emerging role in secondary metabolite biosynthesis under physiological stress (Liu et al. 2025; Li et al. 2024b; Xu et al. 2019). Evidence suggests that PEG-induced drought can alter DNA methylation patterns in *S. rebaudiana*, thereby activating or repressing genes involved in phenolic and flavonoid production and regulating stress-related and metabolite-associated gene networks, contributing to shifts in secondary metabolite accumulation (Zhao et al. 2023; Hajihashemi and Sofo 2018). Interestingly, PEG-induced drought stress has been shown to reduce plant growth while simultaneously inducing the accumulation of secondary metabolites (Zhang and Zhu 2025; Zhou et al. 2022; Li et al. 2024a). This response makes PEG treatment a valuable approach for metabolic engineering in *S. rebaudiana*, enhancing the biosynthesis of bioactive compounds, such as SGs, and promoting the production of other antioxidant metabolites (Lahijanian et al. 2023; Xu et al. 2022; Bai et al. 2025). The above evidence provides the baseline to understand the potential of DNA methylation in secondary metabolite biosynthesis under abiotic stress. Despite this, the underlying mechanism through which DNA methylation regulates SGs production remains poorly understood (Bai et al. 2025). A detailed explanation of PEG-driven metabolic shifts in *S. rebaudiana* is shown in Fig. 5.

## Regulation of SG biosynthesis and their associated genes under PEG-induced drought stress

**Fig. 6:**
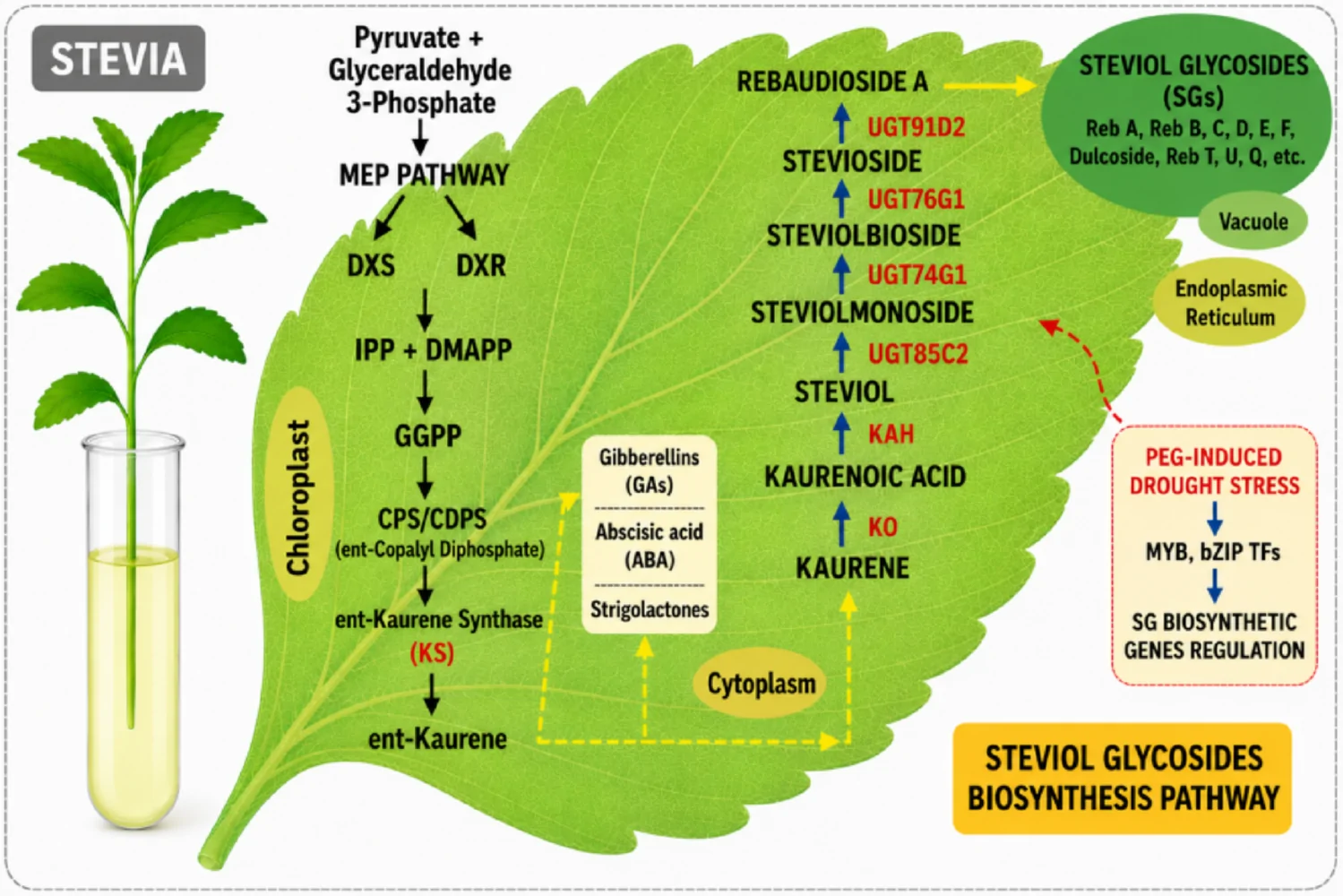
Schematic illustration of the steviol glycosides (SGs) biosynthesis network in *Stevia rebaudiana*, demonstrating the 2-C-Methyl-D-erythritol 4-phosphate (MEP) pathway and the key enzymes that mediate each step of SG synthesis

Stevia synthesizes a diverse group of eight diterpene glycoside compounds in its leaves, derived from the ent-kaurene diterpenoid pathway (Singh and Rao 2005). It is involved in the metabolic transformation of simple diterpenoid precursors into sweet-flavored SGs (Basharat et al. 2021). Among the SGs, stevioside and rebaudioside A are the principal diterpenoid markers of stevia with sweetening characteristics, while others, such as rebaudioside-B, C, D, E, and F, and dulcoside are less sweet (Woelwer-Rieck et al. 2010). These compounds are synthesized via MEP pathway, which provides the diterpenoid precursors vital for the SG biosynthesis. The MEP pathway represents the initial stage of SG production and it has been reported that about 15 enzymes are involved in the overall SG biosynthesis pathway, many of which play essential roles in SG production, and any of them may act as a limiting factor (Kumar et al. 2012a, b). Among these, 1-deoxy-D-xylulose 5-phosphate synthase (DXS) and 1-deoxy-D-xylulose-5-phosphate reductoisomerase (DXR) are regarded as key regulators of the MEP pathway, play critical roles in SG biosynthesis (Fan et al. 2016; Lucho et al. 2018). In addition to its role in SG biosynthesis, MEP pathway is largely linked with plant growth and its development, including the biosynthesis of phytohormones such as GAs, ABA, and strigolactone (Cordoba et al. 2009). Therefore, DXS has also been recognized as a key modulator for the biosynthesis of isoprenoid hormones such as ABA and GA (Kumar et al. 2012a). Downstream of the MEP pathway, ent-copalyl diphosphate synthase (CDPS/CPS) and kaurene synthase (KS) catalyze key reactions leading to the formation of ent-kaurene, an important intermediate shared by both the SG and GA biosynthetic pathways. Therefore, factors that influence the expression or activity of DXS, DXR, CPS, and KS may alter metabolic flux through the pathway and consequently affect SG biosynthesis (Kumar et al. 2012b). A detailed representation of MEP pathway along with the enzymes involved in the conversion of precursor molecules into steviol and SGs, is presented in Fig. 6.

The final step in steviol glycoside formation is a cytosolic glycosylation cascade mediated by UDP-dependent glycosyltransferases (UGTs). Key enzymes involved in the synthesis of SGs include UGT85C2, UGT74G1, UGT76G1, and UGT91D2 (Libik-Konieczny et al. 2020). UGT85C2 initiates steviolmonoside formation, UGT91D2 contributes to rebaudioside diversification by converting Reb A to Reb D, and several rare SGs (Reb T, U, and Q) have recently been identified, although their sweetening properties remain poorly understood (Zhang et al. 2020). The biosynthesis of SGs is closely coordinated by multiple metabolic pathways and enzymes and is sensitive to environmental stresses, such as PEG-induced drought stress, which modulates SG production by restricting water availability and altering key regulatory steps in the pathway (Libik-Konieczny et al. 2021). Studies have demonstrated that PEG-modulated drought stress affects the biosynthesis of SGs in stevia leaves (Pal et al. 2023; Ahmad et al. 2020; Ghaderi et al. 2024). Moreover, PEG induced stress-responsive genes involved in abiotic stress, including MYB and bZIP transcription factors, which are involved in environmental stress tolerance. Such transcription factors can directly or indirectly modulate the biosynthesis of SG related genes (Sun and Fernie 2024; Hajihashemi and Geuns 2016), indicating that PEG stress activates the molecular mechanisms necessary for SGs biosynthesis. The findings of Khan et al. (2024) demonstrated a significant enhancement of steviol glycoside (SG) accumulation under PEG-induced drought stress, highlighting the potential of controlled osmotic stress as an elicitation approach to stimulate SG biosynthetic pathways. The response of SGs to PEG-induced stress in *S. rebaudiana* is complex and not always uniform; while many studies indicate an increase in accumulation under moderate water stress, other research, such as that by (Magangana et al. 2021), reports reductions in SG levels. This divergence can be attributed to multiple factors, including the severity and duration of the stress, the specific stevia genotype, the cultivation conditions (in vitro vs. field), the methodology employed, as well as changes in the individual profile of SGs. These contrasting responses at the molecular level reflects in the transcriptional regulation of SG biosynthesis genes under PEG-induced drought stress. Moreover, the response of stevia towards abiotic stress largely differs according to the experimental system used in the study (Debnath et al. 2019). For instance, a study demonstrated a significantly different response between SG accumulation and biosynthetic activity in vitro cultures and intact plants (Kumari and Chandra 2015). Such variations might be because of the lower degree of tissue differentiation in callus and cell suspension cultures, while the formation of functional chloroplast considered to play a key role in efficient SG biosynthesis (Bondarev et al. 2019; Ceunen and Geuns 2013b). Furthermore, gene expression analyses under PEG-induced drought stress confirm that SG biosynthetic genes exhibit differential regulation on stress intensity and genotypes. Previous study has demonstrated a non-uniform expression of key pathway genes under PEG stress (Tavakoli et al. 2019). Other studies reported similar variability in MEP pathway genes, including DXS and DXR under drought conditions (Lucho et al. 2018; Hajihashemi et al. 2013). Although, the precise mechanism by which PEG enhances SG accumulation in stevia remains incompletely understood. Therefore, future studies must delve deeper into identifying the optimal stress conditions to selectively maximize the production of the most valuable SGs, contributing to more effective cultivation strategies. The SG biosynthetic pathway has gained considerable research attention, largely because it intersects with the GA pathway, sharing four key intermediates fundamental to plant growth and development (Singh et al. 2017).

Taken together, the current evidences demonstrate that SG biosynthesis is strongly associated with metabolic pathways, sequential glycosylation reactions, and stress-responsive transcriptional networks. All of these are greatly affected by PEG-induced drought stress. Although PEG has shown potential as a biological elicitor to enhance SG production, a detailed investigation focusing on PEG-induced drought stress effects on SG accumulation and its associated gene responses in stevia leaves remains insufficiently investigated. Therefore, expanding molecular insights of PEG-induced stress becomes crucial for reducing ROS-mediated oxidative damage and maximizing SG production in *S. rebaudiana*. This advancement will create opportunities for stevia's sustainability while augmenting it’s both commercial and medicinal uses.

## Conclusion and future aspects

*S. rebaudiana* demonstrates a robust adaptive response to PEG-induced drought stress, which affects its morpho-physiological, biochemical, agronomic, and metabolic pathways. PEG treatment enhances drought tolerance while simultaneously stimulating antioxidant and secondary metabolite production and SG biosynthesis, underscoring the strong linkage between stress-driven metabolic reprogramming and plant resilience. Continued molecular studies, particularly those targeting transcriptional regulation, epigenetic modification, and flux through key biosynthetic routes, are essential for elucidating the mechanisms that govern these responses. Furthermore, integrating advanced biotechnological approaches, such as CRISPR 9 (Cas9)-based genome editing, transcriptomic analysis, and metabolomic profiles, will be pivotal for developing improved *S. rebaudiana* genotypes with greater stress tolerance and enhanced bioactive compounds.
